# Susceptibility of eight species members in the *Anopheles hyrcanus* group to nocturnally subperiodic *Brugia malayi*

**DOI:** 10.1186/1756-3305-6-5

**Published:** 2013-01-04

**Authors:** Atiporn Saeung, Chayanit Hempolchom, Visut Baimai, Sorawat Thongsahuan, Kritsana Taai, Narissara Jariyapan, Udom Chaithong, Wej Choochote

**Affiliations:** 1 Department of Parasitology, Faculty of Medicine, Chiang Mai University, Chiang Mai, 50200, Thailand; 2 Department of Biology and Centre for Vectors and Vector-Borne Diseases, Faculty of Science, Mahidol University, Bangkok, 10400, Thailand; 3 Faculty of Veterinary Science (Establishment Project), Prince of Songkla University, Songkhla, 90110, Thailand

**Keywords:** *Anopheles hyrcanus* group, *Brugia malayi*, Susceptibility level, Refractory factor, Thailand

## Abstract

**Background:**

Filariasis, caused by *Brugia malayi*, is a public health problem in Thailand. Currently, at least two locations in southern Thailand are reported to be active endemic areas. Two and four *Mansonia* species are primary and secondary vectors, respectively, of the nocturnally subperiodic race, whereas, *Coquillettidia crassipes* is a vector of the diurnally subperiodic race. Although several *Anopheles* species have been incriminated extensively as natural and/or suspected vectors of *B. malayi*, little is known about vector competence between indigenous *Anopheles* and this filaria in Thailand. Thus, the susceptibility levels of eight species members in the Thai *An. hyrcanus* group to nocturnally subperiodic *B. malayi* are presented herein, and the two main refractory factors that affect them in different degrees of susceptibility have been elucidated.

**Methods:**

*Aedes togoi* (a control vector), *An. argyropus*, *An. crawfordi*, *An. nigerrimus*, *An. nitidus*, *An. paraliae*, *An. peditaeniatus*, *An. pursati* and *An. sinensis* were allowed to feed artificially on blood containing *B. malayi* microfilariae, and dissected 14 days after feeding. To determine factors that take effect at different susceptibility levels, stain-smeared blood meals were taken from the midguts of *Ae. togoi*, *An. peditaeniatus*, *An. crawfordi*, *An. paraliae*, *An. sinensis* and *An. nitidus* immediately after feeding, and their dissected-thoraxes 4 days post blood-feedings were examined consecutively for microfilariae and L_1_ larvae.

**Results:**

The susceptibility rates of *Ae. togoi*, *An. peditaeniatus*, *An. crawfordi*, *An. nigerrimus*, *An. argyropus*, *An. pursati*, *An. sinensis*, *An. paraliae* and *An. nitidus* to *B. malayi* were 70–95%, 70–100%, 80–85%, 50–65%, 60%, 60%, 10%, 5%, and 0%, respectively. These susceptibility rates related clearly to the degrees of normal larval development in thoracic muscles, i.e., *Ae. togoi*, *An. peditaeniatus*, *An. crawfordi*, *An. paraliae*, *An. sinensis* and *An. nitidus* yielded normal L_1_ larvae of 93.15%, 96.34%, 97.33%, 23.60%, 15.38% and 0%, respectively.

**Conclusions:**

*An. peditaeniatus*, *An. crawfordi*, *An. nigerrimus*, *An. argyropus* and *An. pursati* were high potential vectors. *An. paraliae* and *An. sinensis* were low potential vectors, while *An. nitidus* was a refractory vector. Two refractory mechanisms; direct toxicity and/or melanotic encapsulation against filarial larval were involved in the refractoriness of development in the thoracic muscles of the mosquito.

## Background

Lymphatic filariasis, due to *Wuchereria bancrofti*, *Brugia malayi* and *B. timori*, is a major health problem in many tropical and sub-tropical countries. At present, 1.3 billion people worldwide are at risk of lymphatic filariasis infection, with approximately 120 million affected in 72 countries [1-4]. In Thailand, at least two endemic areas of lymphatic filariasis have been reported, i.e., *B. malayi* in the south and *W. bancrofti* on the southwest to northwest Thai-Myanmar border [5,6].

So far, at least two physiological races of *B. malayi*, i.e., nocturnally subperiodic and diurnally subperiodic have been discerned in southern Thailand. The nocturnally subperiodic race is located in endemic areas of five provinces, i.e., Nakhon Si Thammarat, Phattalung, Pattani, Yala and Narathiwat. These regions are rural and semi-forested, and *Mansonia uniformis* and *Ma. bonneae* are the primary vectors in open swamp and swamp-forested areas, respectively, whereas *Ma. dives*, *Ma. indiana*, *Ma. annulata* and *Ma. annulifera* are considered as secondary vectors. The endemic area for the diurnally subperiodic race is confined to Surat Thani province, and *Coquillettidia crassipes* is an important vector [6-8]. When comparing these six provinces, Narathiwat is the highest endemic area, with more than half of the filariasis cases reported there each year. This may result from suitable microhabitats or large areas of swamp for *Mansonia* breeding-places; the existence of cats as animal reservoir hosts; or local insurgence that is considered a main factor in bringing about control failure in this province [8,9]. Regarding control measures, the reduction of microfilariae in the peripheral blood of carriers interrupts the mosquito-transmitted cycle by using a microfilaricide (diethylcarbamazine, [DEC]), which was established in 2002 by the Division of Filariasis, Department of Communicable Disease Control, Ministry of Public Health, Thailand. Consequently, the provinces of Surat Thani and Narathiwat are considered active endemic areas of diurnally and nocturnally subperiodic *B. malayi*, respectively [10]. Despite the control program succeeding at satisfactory levels, as determined by the reduction of microfilaraemic cases to 0% in four provinces (Nakhon Si Thammarat, Phattalung, Pattani and Yala), the two active endemic areas (Surat Thani and Narathiwat provinces) are still regarded as a source of microfilaria. The transmitting cycle has the potential to generate infection not only in these two active endemic areas, but also in adjacent provinces, due to migration of microfilaraemic carriers and long-term settlements as well as inadequate control of animal reservoir-hosts. In addition, this endemic disease could re-emerge at any time, even in thoroughly controlled endemic regions, where the environmental factor(s) favors suitable conditions for the transmission-cycle. This was reported recently in other mosquito-borne diseases, e.g., re-emergence of malaria due to *Plasmodium vivax* in South Korea [11-13].

In southern Thailand, only one and six mosquito species, which have been incriminated as natural vectors of *B. malayi*, belong to the genera *Coquillettidia* and *Mansonia*, respectively. Besides, at least one anopheline species of the subgenus *Cellia* (*An. minimus*) and five of the subgenus *Anopheles* (*An. barbirostris*, *An. campestris*, *An. donaldi*, *An. lesteri* and *An. sinensis*) were reported and incriminated as natural and/or suspected vectors of this filarial nematode in southeast and/or east Asian regions [14]. This information clearly emphasizes that knowledge of the vector competence of *Anopheles* mosquitoes to *B. malayi* is lacking, particularly according to data on the susceptibility level of indigenous *Anopheles* species to a local strain of *B. malayi*. Hence, this study reports the susceptibility of eight species members of the indigenous Thai *An. hyrcanus* group (*An. argyropus*, *An. crawfordi*, *An. nigerrimus*, *An. nitidus*, *An. paraliae*, *An. peditaeniatus*, *An. pursati* and *An. sinensis*) to nocturnally subperiodic *B. malayi* (Narathiwat province, southern Thailand strain). Additionally, the possible factor(s) affecting the different degrees in susceptibility of these anopheline species to nocturnally subperiodic *B. malayi* was elucidated.

## Methods

### Mosquito species and strains

As *B. malayi* is endemic, eight species members of the *An. hyrcanus* group were collected mainly in southern Thailand. This location comprised: (1) former endemic provinces [Chumphon (CP) and Nakhon Si Thammarat (NS)], and (2) provinces adjacent to former and/or current endemic provinces [Phang Nga (PG), Songkhla (SK), and Trang (TG)]. In addition, two provinces free from *B. malayi* infection in western [Ratchaburi (RB)] and northeastern [Ubon Ratchathani (UR)] Thailand were included in this study. The species and strains of the *An. hyrcanus* group were as follows: *An. argyropus* (NS strain), *An. crawfordi* (CP and TG strains), *An. nigerrimus* (UR, NS and SK strains), *An. nitidus* (UR and PG strains), *An. paraliae* (RB strain), *An. peditaeniatus* (CP and SK strains), *An. pursati* (RB strain) and *An. sinensis* (CP strain). Wild-caught, fully engorged females of the 8 *An. hyrcanus* species were collected from cow-baited traps and established successfully for many consecutive generations in the insectary of the Department of Parasitology, Faculty of Medicine, Chiang Mai University, Thailand, using the techniques described previously [15,16]. These colonies were used for studies on susceptibility to nocturnally subperiodic *B. malayi* throughout the experiments. Regarding the control vector, autogenous *Ae*. *togoi* (Chanthaburi province, eastern Thailand) was selected as a proven efficient laboratory vector for a wide-range of genera and species of filarial nematodes, including the nocturnally subperiodic *B. malayi *[17,18].

### Nocturnally subperiodic *B. malayi*

This filarial parasite originated from a 20-year-old women, who was a resident of Bang Paw district, Narathiwat province, southern Thailand. Domestic cats were later infected experimentally with the parasite, which was maintained at the Department of Medical Entomology, Faculty of Tropical Medicine, Mahidol University, Bangkok, Thailand, from 1982 to 1986, when it was transferred to Mongolian jirds (*Meriones unguiculatus*) and then maintained at the animal house of the Faculty of Medicine, Chiang Mai University, Chiang Mai, Thailand [19].

### Preparation of blood containing *B. malayi* microfilariae

The jirds were intraperitoneally inoculated for at least 3 months with infective larvae of nocturnally subperiodic *B. malayi *[20] and anesthetized deeply with ethylene ether. The microfilariae were collected by injecting 3 ml of Hank’s Balanced Salt Solution (HBSS, pH 7.2-7.4) into the peritoneal cavity before withdrawing by peritoneal washing. The 0.05 ml of peritoneal-washed-rich microfilariae was mixed with 10 ml of human-heparinized blood (10 units of heparin/ml of blood), taken from human volunteers who had signed the consent form. Then, the adjusted microfilarial density ranged from approximately 200 to 300 microfilariae (mf)/20 μl by using the human-heparinized blood for artificially feeding all of the mosquito species. The reason for adjusting microfilarial density in blood to range from 200 to 300 mf/20 μl was based on several proven experiments that yielded satisfactorily susceptible *Ae. togoi* to nocturnally subperiodic *B. malayi* (susceptibility rates: 70–95%). This agreed with experiments reporting susceptibility of *An. sinensis* to periodic *B. malayi*, i.e., using a microfilarial density of 5, 10, 20 and 50 mf/20 μl, with a susceptibility rate of 30, 65, 93 and 100%, respectively [21].

### Infection of mosquitoes with *B. malayi* microfilariae

Five-day-old adult female *Ae*. *togoi*, *An. argyropus*, *An. crawfordi*, *An. nigerrimus*, *An. nitidus*, *An. paraliae*, *An. peditaeniatus*, *An. pursati* and *An. sinensis* fasted for 24 hrs and then were allowed artificial feeding simultaneously on blood-containing *B. malayi* microfilariae (microfilarial density = 312, 208, 256 and 283 mf/20 μl in experiment 1, 2, 3 and 4, respectively), using the techniques and apparatus previously described [22]. Fourteen days after feeding, all infected mosquitoes were dissected in normal saline solution and examined under a dissecting microscope. The number of mosquitoes with one or more infective stage larvae in any part of the body (head, thorax or abdomen) was recorded.

### Determination of the possible factor(s) affecting the level of susceptibility

Five-day-old adult female mosquitoes, i.e., an efficient laboratory vector (*Ae. togoi*), high potential vectors [*An. peditaeniatus* (CP strain) and *An. crawfordi* (TG strain)], low potential vectors [*An. paraliae* (RB strain) and *An. sinensis* (CP strain)] and a refractory vector [*An. nitidus* (PG strain)] were allowed artificial feeding simultaneously on blood containing *B. malayi* microfilariae, as mentioned above. The infected mosquitoes were divided into 2 groups, i.e., (1) those with their midgut extracted immediately after full engorgement. The ingested blood meals were then made into thick blood films, dried out, de-hemoglobinized, fixed with methanol, stained with Giemsa (pH 7.2) and counted for microfilariae under a compound microscope; and (2) those with their thorax severed, torn in a drop of normal saline solution and examined under a compound microscope 4 days after feeding. The first stage (L_1_) larvae were counted and scored as normal L_1_ larvae if alive with intact morphology. The larvae were scored as melanized L_1_ if they had evidence of a retained stage and melanotic encapsulation; and scored as degenerated L_1_ if they demonstrated vacuolated internal organs without any evidence of melanotic encapsulation.

### Ethical clearance

The protocols were approved by the Animal Ethics Committee of Faculty of Medicine, Chiang Mai University, Chiang Mai, Thailand.

## Results

Details of the infective rates and parasite loads of *Ae. togoi*, *An. argyropus*, *An. crawfordi*, *An. nigerrimus*, *An. nitidus*, *An. paraliae*, *An. peditaeniatus*, *An. pursati* and *An. sinensis* 14 days after feeding on blood containing *B. malayi* microfilariae are shown in Table 1. The 95%, 70%, 80% and 80% infective rates corresponded to an average of 19.05, 7.50, 10.56 and 11.81 infective (L_3_) larvae per infected *Ae. togoi* in experiment 1, 2, 3 and 4, respectively, which indicated that all feeding experiments were under conditions of sufficient *B. malayi* microfilarial densities in infected blood.

**Table 1:** **Infective rates and parasite loads of 8 species in the *An. hyrcanus *group after feeding on blood containing *B. malayi *microfilariae (microfilarial density = 312, 208, 256 and 283 mf/20 μl in experiment 1, 2, 3 and 4, respectively), with all mosquitoes dissected 14 days after feeding**

**Mosquito species**	Infective rates (No.)*	**Average No. L**_**3**_** per infected mosquito (range)**^**+**^	**L**_**3**_**-distribution**		
			**% head (No.)**	**% thorax (No.)**	**% abdomen (No.)**
Experiment 1					
*Ae. togoi*	95 (19/20)	19.05 (1–49)	59.39 (215)	21.27 (77)	19.34 (70)
*An. crawfordi* (CP)	85 (17/20)^a^	6.24 (1–27)^n^	52.83 (56)	38.68 (41)	8.49 (9)
*An. nigerrimus* (NS)	65 (13/20)^b^	9.77 (1–32)^o^	44.88 (57)	38.58 (49)	16.54 (21)
*An. nigerrimus* (SK)	65 (13/20)^c^	6.69 (1–15)^p^	45.98 (40)	19.54 (17)	34.48 (30)
*An. nitidus* (PG)	0 (0/20)^d^	-	-	-	-
Experiment 2					
*Ae. togoi*	70 (14/20)	7.50 (1–36)	83.81 (88)	9.52 (10)	6.67 (7)
*An. argyropus* (NS)	60 (12/20)^e^	2.92 (1–6)^q^	68.57 (24)	22.86 (8)	8.57 (3)
*An. nigerrimus* (UR)	50 (10/20)^f^	4.20 (1–9)^r^	69.05 (29)	16.66 (7)	14.29 (6)
*An. nitidus* (UR)	0 (0/20)^g^	-	-	-	-
*An. pursati* (RB)	60 (12/20)^h^	3.83 (1–11)^s^	67.39 (31)	19.57 (9)	13.04 (6)
Experiment 3					
*Ae. togoi*	80 (16/20)	10.56 (1–32)	73.37 (124)	16.57 (28)	10.06 (17)
*An. paraliae* (RB)	5 (1/20)^i^	1.00 (0–1)^t^	100.00 (1)	-	-
*An. peditaeniatus* (CP)	100 (20/20)^j^	7.75 (1–23)^u^	57.42 (89)	14.19 (22)	28.39 (44)
Experiment 4					
*Ae. togoi*	80 (16/20)	11.81 (2–28)	77.25 (146)	15.87 (30)	6.88 (13)
*An. crawfordi* (TG)	80 (16/20)^k^	6.06 (1–19)^v^	79.38 (77)	11.34 (11)	9.28 (9)
*An. peditaeniatus* (SK)	70 (14/20)^l^	8.00 (2–22)^w^	80.36 (90)	10.71 (12)	8.93 (10)
*An. sinensis* (CP)	10 (2/20)^m^	1.50 (1–2)^x^	100.00 (3)	-	-

*Fisher exact test: a, j, k vs. control, *P* > 0.05; b, c vs. control, *P* < 0.05.

*Chi-square test: e, f, h, l vs. control, *P* > 0.05; d, g, i, m vs. control, *P* < 0.05.

^**+**^*t*-test (two-sided); q, r, s, u, w vs. control: *P* > 0.05; n, o, p, t, v, x vs. control: *P* < 0.05.

The infective rates (IR) and average number of L_3_ larvae per infected mosquito (AL_3_) of *An. crawfordi* [experiment 1 (CP strain: IR = 85%, AL_3_ = 6.24) and experiment 4 (TG strain: IR = 80%, AL_3_ = 6.06)], *An. nigerrimus* [experiment 1 (NS strain: IR = 65%, AL_3_ = 9.77; SK strain: IR = 65%, AL_3_ = 6.69) and experiment 2 (UR strain: IR = 50%, AL_3_ = 4.20)], *An. nitidus* [experiment 1 (PG strain: IR = 0%, AL_3_ = 0%) and experiment 2 (UR strain: IR = 0%, AL_3_ = 0%)], *An. argyropus* [experiment 2 (NS strain: IR = 60%, AL_3_ = 2.92)], *An. pursati* [experiment 2 (RB strain: IR = 60%, AL_3_ = 3.83)], *An. paraliae* [experiment 3 (RB strain: IR = 5%, AL_3_ = 1.00)], *An. peditaeniatus* [experiment 3 (CP strain: IR = 100%, AL_3_ = 7.75) and experiment 4 (SK strain: IR = 70%, AL_3_ = 8.00)] and *An. sinensis* [experiment 4 (CP strain: IR = 10%, AL_3_ = 1.50)] were mostly lower than those in *Ae. togoi*, an efficient control vector. This was the case in all experimental studies, except for the infective rate of *An. peditaeniatus* (100%), which was higher than that of *Ae. togoi* (80%) in experiment 3. Comparative statistical analyses of the infective rates and average number of L_3_ larvae per infected mosquito were carried out between *Ae. togoi* and all *An. hyrcanus* species. The results revealed that the infective rates between *Ae. togoi* and *An. hyrcanus* species in experiment 1 [*An. crawfordi* (CP strain)], 2 [*An. argyropus* (NS strain), *An. nigerrimus* (UR strain) and *An. pursati* (RB strain)], 3 [*An. peditaeniatus* (CP strain)] and 4 [*An. crawfordi* (TG strain) and *An. peditaeniatus* (SK strain)], and average number of L_3_ larvae per infected mosquito between *Ae. togoi* and *An. hyrcanus* species in experiment 2 [*An. argyropus* (NS strain), *An. nigerrimus* (UR strain) and *An. pursati* (RB strain)] and 3 [*An. peditaeniatus* (CP strain) and 4 (SK strain)] did not differ significantly (*P* > 0.05). It is noteworthy that all infective larvae obtained from the four experimental feedings were very active and found to distribute in all regions of the head, thorax and abdomen, and their behavior was similar, with more than 44% of infective larvae migrating from the thorax to the head and proboscis.

**Figure 1:**
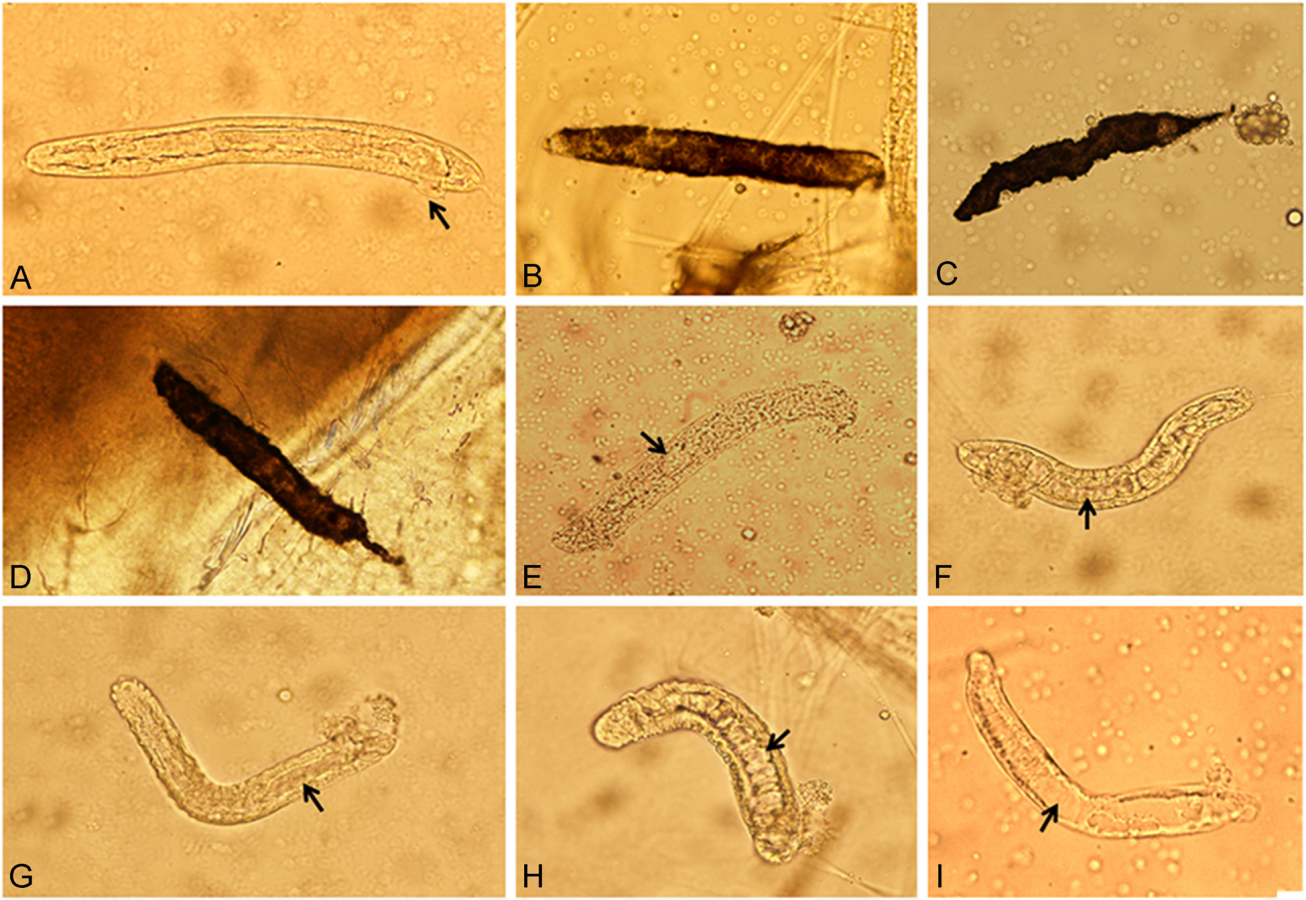
**L_1_ larvae recovered from thoracic muscles of mosquitoes 4 days after infected blood meals.** (**A**) Normal live larva with intact cuticle and internal organs (small arrow: protuberance of anal plug at the anal pore) recovered from *Ae. togoi*. (**B**, **C**, **D**) Completely melanotic encapsulated larvae obtained from *An. paraliae*, *An. sinensis* and *An. nitidus.* (**E**, **F**, **G**, **H**, **I**) Degenerated and vacuolated internal organs (small arrow) acquired from *An. peditaeniatus*, *An. crawfordi*, *An. sinensis, An. paraliae* and *An. nitidus*, respectively.

Parasite loads dissected immediately and 4 days after feeding on blood containing *B. malayi* microfilariae in *Ae. togoi*, *An. peditaeniatus*, *An. crawfordi*, *An. paraliae*, *An. sinensis* and *An. nitidus* are detailed in Table 2. Investigative results on stain-smeared blood meals from the midguts of mosquitoes fed immediately, and fully engorged, indicated that all the mosquito species were successful in taking a considerable number of microfilariae from infected blood, with an average number of microfilariae per infected midgut of 25.20, 27.40, 22.00, 32.40, 26.40 and 25.80 in *Ae. togoi*, *An. peditaeniatus*, *An. crawfordi*, *An. paraliae*, *An. sinensis* and *An. nitidus*, respectively. Likewise, a satisfactory average number of 14.60, 16.40, 15.00, 17.80, 15.60 and 10.80 L_1_ larvae were recovered in the thoracic muscles of *Ae. togoi*, *An. peditaeniatus*, *An. crawfordi*, *An. paraliae*, *An. sinensis* and *An. nitidus*, respectively. However, variations in degrees of normal and abnormal L_1_ larval development in the thoracic muscles of six mosquito species were observed clearly. *Ae. togoi*, *An. peditaeniatus*, *An. crawfordi*, *An. paraliae*, *An. sinensis* and *An. nitidus* yielded normal, melanized and degenerated L_1_ larvae of 93.15%, 0% and 6.85%; 96.34%, 0% and 3.66%; 97.33%, 0% and 2.67%; 23.60%, 47.19% and 29.21%; 15.38%, 32.05% and 52.57%; 0%, 94.44% and 5.56%, respectively (Figure 1).

**Table 2:** **Parasite loads in *Ae. togoi, An. peditaeniatus, An. crawfordi, An. paraliae, An. sinensis *and *An. nitidus *dissected immediately and 4 days after feeding on blood containing *B. malayi *microfilariae (microfilarial density = 247 mf/20 μl)**

**Mosquito species**	Average No. mf per infected midgut (range)*	**Average No. L**_**1**_** per infected thorax (range)**^**+**^	**% normal L**_**1**_** (No.)**	**% melanized L**_**1**_** (No.)**	**% degenerated L**_**1**_** (No.)**
*Ae. togoi*	25.20 (26–65)	14.60 (14–30)	93.15 (68)	0 (0/73)	6.85 (5)
*An. peditaeniatus* (CP)	27.40 (22–94)	16.40 (12–47)	96.34 (79)	0 (0/82)	3.66 (3)
*An. crawfordi* (TG)	22.00 (15–41)	15.00 (8–25)	97.33 (73)	0 (0/75)	2.67 (2)
*An. paraliae* (RB)	32.40 (20–37)	17.80 (10–17)	23.60 (21)	47.19 (42)	29.21 (26)
*An. sinensis* (CP)	26.40 (23–72)	15.60 (13–36)	15.38 (12)	32.05 (25)	52.57 (41)
*An. nitidus* (PG)	25.80 (13–29)	10.80 (5–11)	0 (0/54)	94.44 (51)	5.56 (3)

*Dissected from 5 midguts; ^+^ Dissected from 5 thoraxes.

## Discussion

To incriminate a mosquito vector in an endemic area of filariasis, it is necessary to confirm the susceptibility rate in a laboratory-bred, clean mosquito colony, which has been fed on carrier blood containing microfilariae. By using this criterion, the susceptibility test in an experimental laboratory is an efficient classical tool when suspecting the potential vector of a certain mosquito species. Nonetheless, susceptibility alone does not imply an important role in the transmission of disease in nature, whereas a refractory one can rule out the significance of a vector entirely [14].

Investigation on the susceptibility of eight species members of the Thai *An. hyrcanus* group to nocturnally subperiodic *B. malayi* indicated that *An. peditaeniatus*, *An. crawfordi*, *An. nigerrimus*, *An. argyropus* and *An. pursati* were high potential vectors. *An. paraliae* and *An. sinensis* were low potential vectors, while *An. nitidus* was a refractory vector. However, a crucial question regarding the susceptibility level determined in this study might be raised, due to the artificial feeding of mosquitoes on blood containing *B. malayi* microfilariae, which was not as natural as direct feeding on cat- and/or jird-infected *B. malayi*. Nevertheless, previous reports [23] confirmed that these two feeding techniques could be used robustly for routine screening of potential mosquito vectors of filarial parasites, since they did not differ significantly. This was despite the artificial feeding technique yielding slightly higher infective rates and parasite loads than the direct feeding method, presumably due to the effect of anticoagulant (10 units of heparin/1 ml of blood).

Among the five high potential vectors, *An. peditaeniatus*, *An. crawfordi* and *An. nigerrimus* were found to be abundant and widely distributed in Thailand and other countries [India (Assam, Bihar and Punjab), Sri Lanka, Bangladesh, China (Hainan Island), Myanmar, Cambodia, Vietnam, Malaysia (Malaysian Peninsular, Sabah and Sarawak), Indonesia (Java and Sumatra) and Brunei], and were proven as outdoor-biters of humans in certain localities of Thailand [24,25]. Regarding vector competence, *An. peditaeniatus* and *An. nigerrimus* have been incriminated so far as suspected vectors of *P. vivax* in Thailand [26-28], as well as *An. nigerrimus* as a potential natural vector of *W. bancrofti* in Phang Nga province, southern Thailand [8], and *An. peditaeniatus* as a secondary vector of Japanese encephalitis virus in China and India [29,30]. Beneficial results reported herein emphasize the potential role of *An. peditaeniatus*, *An. crawfordi*, *An. nigerrimus*, *An. argyropus* and *An. pursati* in transmitting nocturnally subperiodic *B. malayi* in southern Thailand as well as other countries, in which these anopheline species and filarial parasite were found sympatrically and/or co-endemic with malaria and Japanese encephalitis. The list of these potential vector-species could be used as a promising guideline for the field approach to incriminate natural vectors in endemic areas of Brugian filariasis. Remarkably, *An. sinensis* has been incriminated as an important vector of nocturnally periodic *B. malayi* in China, Korea and Japan [14], but in this study, it was proven as a low potential vector of nocturnally subperiodic *B. malayi*. It is interesting to note that the *An. sinensis* strain from Korea and China was compatible genetically and/or nearly identical to that from Thailand, based on the crossing experiments and comparative sequence analyses of the ribosomal DNA (rDNA) internal transcribed spacer 2 (ITS2), and mitochondrial cytochrome *c* oxidase subunit I (COI) and subunit II (COII) [31]. This evidence appeared to support the high specificity between *B. malayi* physiological races and the *An. sinensis* vector.

It has been known for refractoriness of certain mosquito species towards filarial parasites to occur in the forgut (cibarial and pharyngeal amartures), midgut (fast blood coagulation) or thoracic muscles (direct toxicity and melanotic encapsulation) [32-34]. Regarding refractoriness in the thoracic muscle, large numbers of *B. malayi* and *B. pahangi* microfilariae exsheathed in refractory *Ae. albopictus* after gaining entry into the mosquitoes, and subsequently migrated to the thoracic muscles without further development [35,36]. The results revealed that the factor(s) in the thoracic muscles of *Ae. albopictus* conferred with the refractoriness. Evidence of refractoriness to *B. pahangi* microfilariae infection is of additional interest, as it could be induced in normally susceptible *Ae. tabu* by rearing female mosquitoes on sugar solution containing thoracic homogenate of refractory *Ae. malayansis* mosquitoes [33]. This result agreed with a subsequent study in that the high inhibition of *B. pahangi* larval development could be induced in the thoracic muscle of susceptible *Ae. togoi*. This was performed by intrathoracic injection of crude thoracic homogenate (CTH) from refractory *Ae. albopictus* into susceptible *Ae. togoi* prior to feeding on blood containing *B. pahangi* microfilariae [37]. Thus, these two pieces of evidence seem to reflect the inhibitory effect that might be due to direct toxicity of the homogenate on developing larvae. Furthermore, the melanization of immune responses in various insects against a wide-range of invading pathogens and parasites has been documented [34,38-40]. The immune response of mosquitoes is put into effect through the plasma components of both the hemolymph, i.e., the humoral response, and hemocytes, the cellular response [34]. The authors also suggested that the intracellular melanotic encapsulation of filarial developing stages, as observed in specific mosquito organs, may be caused by exposure to low-molecular-weight immune molecules, which are carried in the hemolymph (plasma) and can penetrate the basement membrane covering the cells of specific organs. This concept suggested that the same mechanisms controlling melanotic encapsulation reactions (immune response) extracellularly in the hemocoel also control them intracellularly in specific organs of the host in which the parasite develops. Subsequent evidence from using RNAi methodology to knock-down PAH (phenylalanine hydroxylase) expression in the mosquitoes, *Ae. aegypti* and *Armigeres subalbatus*, demonstrated that limitation in the amount of tyrosine, available for tyrosinase-mediated hydroxylation, significantly reduces the effectiveness of melanization reactions against inoculated filarial parasites [41]. Additionally, at least four specific enzymes [DCE (dopachrome conversion enzyme), DDC (dopa decarboxylase), PO (phenoloxidase) and TH (tyrosine hydroxylase)] were concerned in the biosynthesis of melanin [39]. Current studies on the possible factors affecting the difference in susceptibility levels of eight *An. hyrcanus* species to nocturnally subperiodic *B. malayi* revealed that at least two refractory mechanisms (direct toxicity and/or melanotic encapsulation) were involved in the refractoriness of thoracic muscles for parasite development. Variations in the percentages of melanotic encapsulation and degenerated L_1_ larvae recovered in the thoracic muscles of *Ae. togoi* (0% and 6.85%), *An. peditaeniatus* (0% and 3.66%), *An. crawfordi* (0% and 2.67%), *An. paraliae* (47.19% and 29.21%), *An. sinensis* (32.05% and 52.57%) and *An. nitidus* (94.44% and 5.56%), were good supportive evidence.

## Conclusions

Eight species members of the *An. hyrcanus* group, i.e., *An. argyropus*, *An. crawfordi*, *An. nigerrimus*, *An. nitidus*, *An. paraliae*, *An. peditaeniatus*, *An. pursati* and *An. sinensis* were tested for susceptibility to nocturnally subperiodic *B. malayi*. They were allowed to feed artificially on blood containing *B. malayi* microfilariae, and dissected 14 days after feeding. The susceptibility rates were 70-100%, 80-85%, 50-65%, 60%, 60%, 10%, 5% and 0% in *An. peditaeniatus*, *An. crawfordi*, *An. nigerrimus*, *An. argyropus*, *An. pursati*, *An. sinensis, An. paraliae* and *An. nitidus*, respectively. As determined by levels of susceptibility, results indicated that *An. peditaeniatus*, *An. crawfordi*, *An. nigerrimus*, *An. argyropus* and *An. pursati* were high potential vectors when compared with the control vector, *Aedes togoi*. *An. paraliae* and *An. sinensis* were low potential vectors, while *An. nitidus* was a refractory vector. In order to determine the possible factor(s) affecting different degrees of susceptibility, stained-smears of blood meals from midguts immediately after fully engorged and dissected-thoraxes 4 days post blood-feeding from the control vector (*Ae. togoi*), high potential vectors (*An. peditaeniatus* and *An. crawfordi*), low potential vectors (*An. paraliae* and *An. sinensis*) and refractory vector (*An. nitidus*) were examined for microfilariae and L_1_ larvae, respectively. The results revealed that an appreciable number of microfilaria obtained in the ingested blood meals and L_1_ larvae recovered in thoracic muscles was similar in appearance to those in all infected mosquitoes. Nonetheless, the marked variations in degrees of normal development of L_1_ larvae in thoracic muscles were observed clearly from the four vector-groups, i.e., the control vector: 93.15%, high potential vectors: 96.34–97.33%, low potential vectors: 15.38–23.60% and refractory vector: 0%. At least, two refractory mechanisms, direct toxicity and melanotic encapsulation, were involved in the inhibition of L_1_ larval development in thoracic muscles.

## Competing interests

The authors declare no competing interests.

## Authors’ contributions

All the authors contributed significantly to this study. AS participated in the study design, field and laboratory experiments, data analysis and writing of the manuscript. CH, ST and KT carried out field and laboratory experiments. VB participated in data analysis, and criticized the manuscript. NJ and UC helped with data analyses. WC designed the experiments, carried out field and laboratory experiments, interpreted the results, and edited the manuscript. All authors read and approved the final version of the manuscript.
